# Distinguishing the lipid profile of GCK-MODY patients and its correlation with hsCRP levels

**DOI:** 10.3389/fendo.2022.1024431

**Published:** 2022-10-26

**Authors:** Fan Ping, Junling Fu, Xinhua Xiao

**Affiliations:** Department of Endocrinology, National Health Commission (NHC) Key Laboratory of Endocrinology, Peking Union Medical College Hospital, Chinese Academy of Medical Sciences and Peking Union Medical College, Beijing, China

**Keywords:** maturity-onset diabetes of the young, glucokinase, cardiovascular protection, high-sensitivity C-reactive protein, serum lipid profile

## Abstract

**Aims:**

Glucokinase–maturity-onset diabetes of the young (GCK-MODY) is the most common monogenic diabetes in China. We have previously reported on the low levels of high-sensitivity C-reactive protein (hsCRP) in patients with GCK-MODY. In this study, we further explored the correlation between the serum lipid profiles and hsCRP levels of patients with different types of diabetes. We also proposed to determine the possible mechanism of macrovascular protection in *GCK* genetic variants.

**Methods:**

The serum lipid profiles of the GCK-MODY group (*n* = 50) were compared with those of the hepatocyte nuclear factor-1 alpha (HNF1A)-MODY group (*n* = 19), the type 1 diabetes (T1D) group (*n* = 50), and the type 2 diabetes (T2D) group (*n* = 54). The associations between the lipid compositions and the hsCRP levels in each group were also explored.

**Results:**

Elevated levels of high-density lipoprotein cholesterol (HDL-C) were found in the GCK-MODY group (1.5 ± 0.27) compared with the T1D (1.2 ± 0.47, *p* < 0.01) and T2D (1.3 ± 0.3, *p* < 0.01) groups. On the other hand, a significantly lower LDL-C level (2.4 ± 0.69) in the GCK-MODY group compared with the T1D (2.7 ± 0.72, *p* < 0.01) and T2D (2.9 ± 0.68, *p* < 0.01) groups was also noted. A lower ratio of triglyceride to HDL-C (TG/HDL) and a lower hsCRP level were also found in the GCK-MODY group [TG/HDL = 0.38 (0.25–0.52), hsCRP = 0.2 mg/L (0.16–0.37)] compared with the T1D group [(TG/HDL = 0.56 (0.39–1.29), hsCRP = 0.56 mg/L (0.39–1.29), *p* < 0.01] and the T2D group [(TG/HDL = 1.6 (1.1–2.68), hsCRP = 1.11 mg/L (0.66–2.34), *p* < 0.01]. Although patients with HNF1A-MODY showed similar hsCRP levels [0.17 (0.08–0.52), *p* > 0.05] compared with the patients in the GCK-MODY group, they had higher TG levels [1.01 (0.66–1.76), *p* < 0.05] and TG/HDL ratios [0.84 (0.56–1.31), *p* < 0.05]. Analysis of the correlations between the hsCRP levels and lipid profiles of each group confirmed that the LnhsCRP (natural logarithm-transformed hsCRP level) was positively correlated with the LnTG (natural logarithm-transformed TG) (*r* = 0.352, *p* = 0.011) and the Ln(TG/HDL) ratio (*r* = 0.283, *p* = 0.047) only in individuals with GCK-MODY.

**Conclusions:**

Individuals harboring *GCK* variants have the characteristics of protective lipid profiles manifested as a higher level of HDL-C and a lower level of LDL-C compared with type 1 and 2 diabetes milletus (T1DM and T2DM, respectively) patients. In addition, lower ratios of TG/HDL were found to be associated with the inhibition of secretion of hsCRP, even when adjusted for the HbA1c levels in patients with GCK-MODY. It is suggested that the protective effect of macrovascular complications in GCK-MODY patients might partly be due to their unique lipid profiles associated with the suppression of inflammation.

## Background

It is well known that patients with glucokinase inactive mutations [glucokinase–maturity-onset diabetes of the young (GCK-MODY) or MODY2] rarely develop diabetic complications, especially cardiovascular diseases (CVDs), and typically do not require treatments (1). Despite long-term exposure to hyperglycemia, the prevalence of macrovascular complications in diabetes patients is close to that of non-diabetic controls. A decreasing tendency was even observed in some macrovascular events, such as angina and clinically diagnosed macrovascular disease, in patients with GCK-MODY compared to healthy controls (2). Patients with GCK-MODY exhibit stable elevated fasting hyperglycemia (5.5–8.5 mmol/L), which is considered the major mechanism of protection from vascular events. However, it has also been reported that, in individuals with impaired fasting glucose (IFG), a mild elevation of the fasting blood glucose levels is also modestly associated with an increased risk of CVD (3). We have reported that elevated levels of high-density lipoprotein (HDL)-resident adipose triglyceride lipase and increased activity of choline/ethanolamine phosphotransferase 1 (CEPT1) may contribute to the atheroprotective effect in GCK-MODY compared to that in type 2 diabetes milletus (T2DM) (4).

In this study, based on a GCK-MODY group with a larger sample size and retrospective groups of patients with type 1 diabetes (T1D), type 2 diabetes (T2D), and hepatocyte nuclear factor-1 alpha (HNF1A)-MODY, we aimed to further compare the blood lipid profiles of patients with GCK-MODY with those in other hyperglycemia groups and to explore whether inflammatory processes are associated with the lipid indices.

## Methods

We recruited a total of 50 patients with GCK-MODY identified by genotyping analysis, 19 patients with hepatic nuclear factor 1 alpha (*HNF1A*) gene mutations (HNF1A-MODY), 50 patients with T1D, and 54 patients with T2DM with an onset age <55 years who had long-term follow-up in the outpatient department of Peking Union Medical College Hospital (PUMCH). All participants were of Han Chinese descent. In order to reduce the likelihood of unrecognized MODY in individuals with T1D, in addition to the presentation of the clinical characteristics of T1D, it was required that none of the T1D-specific autoantibodies to glutamic acid decarboxylase (GAD), islet antigen-2 (IA-2), or islet cell autoantibodies (ICAs) has ever been positively detected.

GCK-MODY or HNF1A-MODY was first suggested in non-obese (BMI < 28 kg/m^2^) and in hyperglycemic individuals without T1D autoantibodies but fulfilling any of the following clinical criteria: 1) family history of diabetes in at least two generations with autosomal-dominant inheritance mode; 2) young onset (less than 45 years); and 3) confirmed by genetic analysis. The T2D group in our study included patients with a hyperglycemia onset age <55 years. All individuals were consecutively recruited from the outpatient clinic of endocrinology at PUMCH, Beijing, China, between January 2014 and December 2020. All procedures followed were in accordance with the ethical standards of the Peking Union Medical College Hospital Ethics Committee and with the Helsinki Declaration of 1964, as revised in 2013. Informed consent was obtained from the participants or the guardians.

Direct genomic sequencing or targeted next-generation sequencing (NGS) followed by Sanger sequencing was performed. The exons, the intron–exon boundaries, and the promoter sequences of the *GCK* and *HNF1A* genes were amplified by the polymerase chain reaction and then sequenced bidirectionally on an ABI 3730XL DNA Analyzer (Applied Biosystems, Waltham, MA, USA). The sequences were compared with the reference genomic GCK sequences NM_000162 (*GCK*) and NM_000545.6 (*HNF1A*) using the Human BLAT Search of the University of California Santa Cruz (http://genome.ucsc.edu/cgi-bin/hgBlat). Variants were considered pathogenic based on prediction analysis, published reports, or segregation within the families.

Medical history including sex, age of diabetes milletus onset, and family history were recorded. The body mass index (BMI) was calculated as mass/height^2^. Fasting C-peptide and 2-h postprandial C-peptide were determined by chemiluminescent analysis. Glycated hemoglobin A1c (HbA1c) was measured using dedicated high-performance liquid chromatography. The antibodies to GAD and IA2 were determined using ELISA (Siemens ADVIA Centaur XP, Munich, Germany), and ICAs were measured using indirect immunofluorescence. The levels of high-sensitivity C-reactive protein (hsCRP) were determined using the immunoturbidimetric method on a Beckman AU5800 analyzer (Beckman Coulter Inc., Brea, CA, USA). Patients with hsCRP values >10 mg/L were considered likely to present an acute inflammatory response and were then excluded from further analysis. The levels of plasma glucose, total cholesterol (TC), high-density lipoprotein cholesterol (HDL-C), low-density lipoprotein cholesterol (LDL-C), and triglyceride (TG) were detected with an automatic biochemical analyzer (AU5800; Beckman Coulter Inc., Brea, CA, USA). Δ2-h glucose was used to represent the increment between the fasting and 2-h postprandial glucose concentrations.

### Statistical analysis

All statistical analyses were performed with SPSS (Statistical Package for Social Science) software, version 21.0 (Chicago, IL, USA), and R language (R package, version 4.1.1). Data were presented as the mean ± SD for variables with a normal distribution, median with interquartile range (IQR) for data with non-normal distribution, and frequency with percentages for categorical variables. The chi-squared test was applied for categorical variables. Multiple comparisons were performed using one-way ANOVA with Bonferroni’s *post*-*hoc* test for variables with a normal distribution. Differences in the non-normally distributed data were calculated using the Kruskal–Wallis test or natural logarithmic transformation prior to comparative studies. Bivariate correlations were evaluated using Pearson’s correlation model for normally distributed data and Spearman’s test for skewed variables. Partial correlations were used to control for confounding factors. Heatmaps were generated using R. The levels of statistical significance were accepted at *p* < 0.05 (two-sided).

## Results

Anthropometric variables, the lipid profiles, glucose metabolism, fasting C-peptide levels, and the hsCRP concentrations of each group are presented in **Table 1**.

**Table 1:** General characteristics of each group.

	*N* (F/M)	Onset age (years)	BMI (kg/m^2^)	FBG (mmol/L)	2hPBG (mmol/L)	ΔBG (mmol/L)	FCP (ng/ml)	2hCP (ng/ml)	HbA1c (%)	TG (mmol/L)	TC (mmol/L)	HDL-C (mmol/L)	LDL-C (mmol/L)	TG/HDL-C ratio	hsCRP (mg/dl)
T**1D**	50 (23/27)	19.6 ± 9.2	20.3 ± 3.43	10.0 ± 5.05	15.5 ± 5.82	6.20 (2.65–9.00)	0.23 (0.00–0.44)	0.48 (0.07–0.89)	10.3 ± 3.03	0.75 (0.52–0.97)	4.70 ± 0.81	1.20 ± 0.47^b^	2.70 ± 0.72	0.56 (0.39–1.29)^b^	0.36 (0.24–1.72)
GCK-MODY	50 (28/22)	24.1 ± 14.9^a^	19.3 ± 4.04	6.9 ± 0.65^a^	9.4 ± 2.63^a^	1.90 (0.80–3.15)^a^	0.80 (0.66–1.13)^a^	3.15 (2.14–4.57)^a^	6.5 ± 0.88^a^	0.56 (0.43–0.71)^c^	4.30 ± 0.77	1.50 ± 0.27^a^	2.40 ± 0.69^a^	0.38 (0.25–0.52)^a,c^	0.20 (0.16–0.37)^a^
HNF1A-MODY	19 (10/9)	26.9 ± 13.0^a^	20.7 ± 2.92	9.3 ± 3.66^b^	14.7 ± 4.61^b^	5.35 (2.60–6.30)^b^	0.88 (0.73–1.14)^a^	2.95 (2.06–4.30)^a^	7.7 ± 2.09^ab^	1.01 (0.66–1.76)^b^	4.40 ± 0.96	1.30 ± 0.31	2.50 ± 0.89	0.84 (0.56–1.31)^b^	0.17 (0.08–0.52)
T2D	54 (30/24)	45.3 ± 6.82^a,b,c^	26.9 ± 4.69^a,b,c^	9.4 ± 3.22^b^	14.9 ± 5.70^b^	5.47 (3.20–8.99)^b^	1.54 (0.96–2.05)^a,b,c^	5.02 (2.97–7.38)^a,b,c^	7.4 ± 1.48^a,b^	1.91 (1.35–3.02)^a,b,c^	5.60 ± 1.14^a,b,c^	1.30 ± 0.30^b^	2.90 ± 0.68^b,c^	1.60 (1.10–2.68)^a,b,c^	1.11 (0.66–2.34)^b,c^

Data were expressed as N for categorical variables, mean ± SD for normally distributed variables, and median (interquartile range) for variables with skewed distributions.

F/M, female/male; BMI, body mass index; TC, total cholesterol; TG, triglycerides; HDL-C, high-density lipoprotein cholesterol; LDL-C, low-density liphoprotein cholesterol; FBG, plasma fasting blood glucose; 2hPBG, 2-h postprandial blood glucose; ΔBG, increment from FBG to 2hPBG; HbA1c, glycosylated hemoglobin; FCP, fasting C-peptide; 2hCP, 2-h postprandial C-peptide; T1D, type 1 diabetes; T2D, type 2 diabetes; GCK, glucokinase; MODY, maturity-onset diabetes of the young; HNF1A, hepatocyte nuclear factor-1 alpha.

Significant discrimination indices are indicated as follows.

a Significant discrimination index, versus T1D patients.

b Significant discrimination index, versus GCK-MODY patients.

c Significant discrimination index, versus HNF1A-MODY patients.

The gender ratio was balanced in all the study groups. The average onset age of patients in the T1D group (19.6 ± 9.20 years) was earlier than that of patients in the GCK-MODY (24.1 ± 14.95 years) and HNF1A-MODY (26.9 ± 13.00 years) groups, while the T2D group had a relatively older age of onset (45.3 ± 6.82 years). It was also found that patients in the T2D group had a higher BMI, but there were no significant differences among those in the GCK-MODY, HNF1A-MODY, and T1D groups [26.9 ± 4.69 kg/m^2^ (T2D) *vs*. 19.3 ± 4.04 kg/m^2^ (GCK-MODY), 20.3 ± 3.43 kg/m^2^ (T1D), and 20.7 ± 2.92 kg/m^2^ (HNF1A-MODY)]. Compared with patients in the other groups, those in the GCK-MODY group showed chronic mild hyperglycemia, including fasting glucose levels [6.9 ± 0.65 mmol/L (GCK-MODY) *vs*. 10.0 ± 5.05 mmol/L (T1DM), 9.4 ± 3.22 mmol/L (T2D), and 9.3 ± 3.66 mmol/L (HNF1A-MODY)], 2-h postprandial glucose levels [9.4 ± 2.63 mmol/L (GCK-MODY) *vs*. 15.5 ± 5.82 mmol/L (T1DM), 14.9 ± 5.70 mmol/L (T2D), and 14.7 ± 4.61 mmol/L (HNF1A-MODY)], postprandial blood glucose elevations [1.9 (0.8–3.15) mmol/L (GCK-MODY) *vs*. 6.2 (2.65–9.00) mmol/L (T1DM), 5.35 (2.6–6.3) mmol/L (HNF1A-MODY), and 5.47 (3.2–8.99) mmol/L (T2D)], and HbA1c [6.5 ± 0.88% (GCK-MODY) *vs*. 10.3 ± 3.03% (T1DM), 7.4 ± 1.48% (T2D), and 7.7 ± 2.09% (HNF1A-MODY)]. There were no differences between the GCK-MODY and HNF1A-MODY groups in terms of the C-peptide levels and hsCRP concentrations, which correspond to the marker of endogenous insulin secretion and the inflammation status, respectively.

It was found that the levels of TG and TC and the ratio of TG/HDL-C in the T2D group were significantly higher than those of the other groups. The LDL-C level, TG/HDL-C ratio, and the hsCRP level in the GCK-MODY group were lower than those of patients with T1D, whereas the HDL-C level was significantly higher than those in the T1D and T2D groups. The lipid profiles in the GCK-MODY group exhibited similar characteristics to those in the HNF1A-MODY group, except for the lower TG levels and TG/HDL ratios.

**Figure 1:**
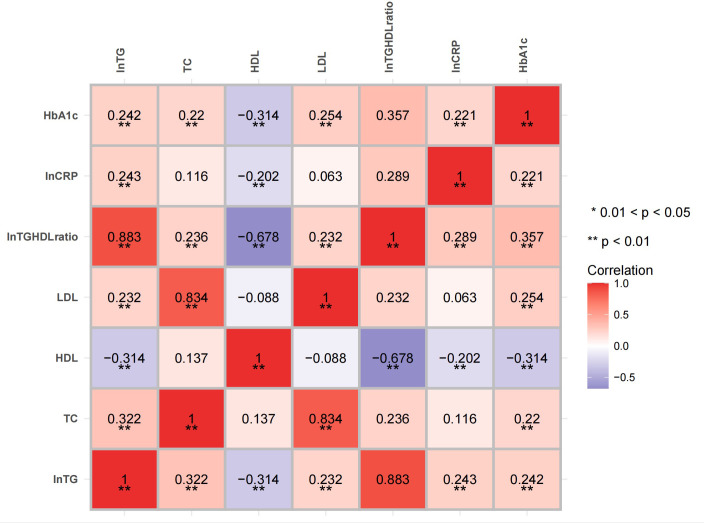
Heatmap of the correlation of glycolipid metabolism and inflammation marker. The correlation between HbA1C, lipid profiles, and the high-sensitivity C-reactive protein (hsCRP) levels adjusted for age and sex. The non-normally distributed traits such as hsCRP, triglyceride (TG), and the TG/HDL (high-density lipoprotein) ratio were natural logarithmic transformed. *Red* and *blue* indicate a positive and a negative association, respectively. *Color intensity* represents the strength of the correlation. *0.01 < *p* < 0.05; **p < 0.01.

**Figure 2:**
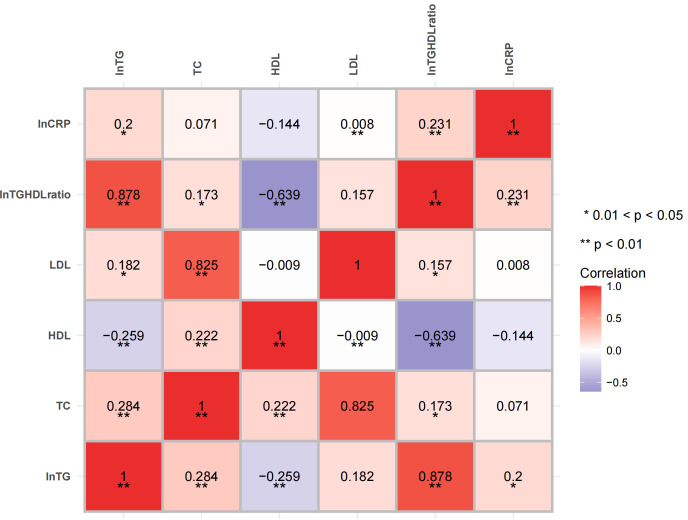
Heatmap of the correlation of the lipid profiles and inflammation marker after adjusting for glycated hemoglobin A1c (HbA1c). The correlations between the lipid profiles and the high-sensitivity C-reactive protein (hsCRP) levels were adjusted for age, sex, and HbA1c. *Red* and *blue* indicate a positive and a negative association, respectively. *Color intensity* represents the strength of the correlation. *0.01 < *p* < 0.05; **p < 0.01.

The correlation analysis showed that both the natural logarithm-transformed TG (LnTG; *r* = 0.243, *p* = 0.002) and the Ln(TG/HDL) ratio (*r* = 0.289, *p* < 0.001) were positively associated with the LnCRP in all individuals after adjusting for age and sex. Positive correlations between LnCRP and HbA1c (*r* = 0.221, *p* = 0.005) were also found (**Figure 1**). After controlling for the HbA1c levels, as well as age and sex, the positive correlations between LnCRP and Ln(TG/HDL) still existed (*r* = 0.231, *p* = 0.003) (**Figure 2**). Subgroup analysis showed that these positive correlations were only present in the GCK-MODY group (**Supplementary 1**). The LnCRP and HDL-C levels were negatively correlated in all individuals (*r* = −0.202, *p* = 0.01), but the association was no longer significant after adjusting for the confounding factors mentioned above.

## Discussion

Dyslipidemia acts as a marker for the development of atherosclerosis, which is the primary cause of CVD and usually occurs in the prediabetic stage. We reported in a previous study that the characteristics of the lipid profiles of the GCK-MODY group are significantly distinct from those of T2D with increased Phosphatidylcholine(PC) and its derivatives (plasmalogen PC) form a class of phospholipids,whch are major components of biological membranes and also the essential component of the very low-density lipoprotein (VLDL) complex. levels (4). In this study, the lipid profiles of the GCK-MODY group were confirmed to have favorable characteristics for macrovascular disease, with significantly lower levels of TG, TC, and LDL-C and higher levels of HDL-C compared with those of the T2D group in which statins were widely used. Patients with inactive GCK mutations were found to have significantly higher HDL-C levels than T1D patients with an earlier age of onset. Our previous study based on a relatively small sample size has already identified lower hsCRP levels in those with GCK-MODY than in T1D individuals with similar BMI (5). The hsCRP level is the most widely used inflammatory biomarker for predicting cardiovascular events independently (6). It has been reported that an extremely suppressed hsCRP level has a discriminative value in the diagnosis of HNF1A-MODY because *HNF1A* is required for the cytokine-driven CRP expression (7). According to a previous study, HNF1A-MODY patients showed an increased IMT (carotid artery intima–media thickness) and a decreased FMD (brachial artery flow-mediated dilatation) in comparison to controls and GCK-MODY individuals of similar age (8). This implies that the lower hsCRP level per se is not a unique cardiovascular protective factor in patients with HNF1A-MODY. Our study found that patients with GCK-MODY had similar hsCRP values to those in the HNF1A-MODY group, which evidenced that the inflammatory response was also suppressed. Nevertheless, this was mediated by different mechanisms. As aforementioned, the TG levels and the TG/HDL ratios were increased in the HNF1A-MODY group compared with the GCK-MODY group.

Therefore, we speculate that there may be a correlation between inflammation and lipid metabolism in patients with GCK-MODY, which may underline the mechanism of the protective role in the occurrence of diabetes macrovascular complications. The correlation analysis confirmed that the LnCRP level was positively related to the natural logarithm of the TG/HDL-C ratio even after controlling for potential confounding factors such as age, sex, and HbA1c level. In addition, subgroup analysis found that the positive correlation mentioned above only existed in the GCK-MODY group.

It has already been demonstrated that the expression levels of GCK are positively associated with intrahepatocellular lipids and *de novo* lipogenesis (9). This suggests that GCK is deeply involved in both glucose and lipid metabolism. The activity of GCK in hepatocytes is regulated by liver-specific glucokinase regulatory protein (GKRP), which binds to the inactive GCK and induces nuclear localization of GCK–GKRP complexes (10). The polymorphism of GKRP (P446L, rs1260326) with inhibitory protein function has been proven to cause elevated TG and CRP levels and, paradoxically, a reduction in fasting glucose mediated by increased GCK activity (11, 12). The Action for Health in Diabetes (Look AHEAD) Study also indicated that T2DM patients with the P446L GKRP variant are resistant to the protective effect of an intensive lifestyle intervention, although moderate improvements in adiposity and fitness were achieved (12). It has also been confirmed *in vitro* that the oxidative damage of β cells induced by extracellular hyperglycemia was caused by enhanced GCK activity (13).

Therefore, we hypothesized that GCK inactivating mutation may confer protective effects against atherosclerosis through the following mechanisms. Firstly, a decrease in the enzyme activity of GCK will lead to the increase of blood glucose levels through upregulating glucose-stimulated insulin secretion (GSIS) in β cells. However, the elevation of blood glucose fails to promote oxidative stress in individuals harboring inactive GCK variants. As a result, β cells are protected from glucose toxicity for a long period without progressive elevation of blood glucose, and these individuals often present with stable fasting mild hyperglycemia. Secondly, the GCK loss-of-function mutation can induce a change in the lipid profile, which may also be involved in the mechanism of the protective role in CVD.9 It was confirmed in the Rotterdam Study that both total and individual intakes of polyunsaturated fatty acids (PUFAs) are negatively correlated with serum CRP levels (14). Our previous study reported elevated PUFA levels in isolated HDLs from patients with GCK-MODY mediated with enhanced adipose triglyceride lipase (ATGL) and choline/ethanolamine phosphotransferase 1 (CEPT1), which may further promote the atheroprotective effect.4 This result raises concern about the side effects on cardiovascular events with long-term GCK agonist use in T2DM treatment.

There are some limitations in this study. Firstly, the study was based on a single-center observational cohort and lacked a uniform prospective intervention. Secondly, the patients with T2DM were widely prescribed statins in accordance with diabetes guidelines, but specific data were not available. Thirdly, due to the limitations of the study design (i.e., except for the T2DM group, most of the participants were young adults or adolescents), we have not routinely evaluated the complications associated with dyslipidemia. In a future study, we will focus on the incidence of macrovascular complications in different groups. Finally, the sample size of the HNF1A-MODY group was relatively small, which was dependent on the specific MODY subtypes prevalent in China.

In conclusion, this study revealed the characteristics of the protective lipid profiles and their association with the inhibition of inflammatory activity in individuals with GCK-MODY. It is also suggested that the above characteristics contribute to the insusceptibility of GCK-MODY patients to CVD.

## Data availability statement

The datasets presented in this study can be found in online repositories. The names of the repository/repositories and accession number(s) can be found in the article/**Supplementary Material**.

## Ethics statement

The studies involving human participants were reviewed and approved by the Peking Union Medical College Hospital Ethics Committee. Written informed consent for participation in this study was provided by the participants’ legal guardian/next of kin.

## Author contributions

FP contributed to the data collection and drafted the manuscript. JF contributed to the data collection and interpretation and reviewed/edited the manuscript. XX was responsible for the concept and design of the study, contributed to the data analysis and interpretation, and revised the manuscript. All authors contributed to the article and approved the submitted version.

## Funding

This research was funded by the CAMS Innovation Fund for Medical Science (CIFMS), grant number 2021-1-I2M-028 and 2021-I2M-C&T-B-010; Diabetes Mellitus Research Fund Program from Shanghai Medical and Health (SHMHDF), grant number DMRFP II_09.

## Acknowledgments

The authors thank the participants for their continuing participation in this research effort.

## Conflict of interest

The authors declare that the research was conducted in the absence of any commercial or financial relationships that could be construed as a potential conflict of interest.

## Publisher's note

All claims expressed in this article are solely those of the authors and do not necessarily represent those of their affiliated organizations, or those of the publisher, the editors and the reviewers. Any product that may be evaluated in this article, or claim that may be made by its manufacturer, is not guaranteed or endorsed by the publisher.
