# Supplementary material for: Distinguishing the lipid profile of GCK-MODY patients and its correlation with hsCRP levels
Source: Front Endocrinol (Lausanne). 2022 Oct 26;13:1024431. doi: 10.3389/fendo.2022.1024431 (PMC9643687; doi:10.3389/fendo.2022.1024431)
Supplement: Supplementary file 1 [file DataSheet_1.docx]

| T1DM | | | | | | | | | | |
| --- | --- | --- | --- | --- | --- | --- | --- | --- | --- | --- |
|  |  | TG | TC | HDL | LDL | TG/HDL | hsCRP | lnTG | lnTG/HDL | lnCRP |
| TG | r | 1.000 | .076 | -.133 | .042 | .184 | -.023 | .902 | .487 | .126 |
|  | p | .000 | .622 | .389 | .788 | .231 | .881 | .000 | .001 | .422 |
| TC | r | .076 | 1.000 | .183 | .853 | .112 | -.056 | .145 | .080 | -.093 |
|  | p | .622 | .000 | .235 | .000 | .471 | .717 | .347 | .606 | .554 |
| HDL | r | -.133 | .183 | 1.000 | -.063 | -.710 | -.119 | -.183 | -.847 | -.159 |
|  | p | .389 | .235 | .000 | .684 | .000 | .444 | .234 | .000 | .310 |
| LDL | r | .042 | .853 | -.063 | 1.000 | .223 | -.104 | .217 | .216 | -.226 |
|  | p | .788 | .000 | .684 | . | .146 | .502 | .157 | .159 | .146 |
| TG/HDL | r | .184 | .112 | -.710 | .223 | 1.000 | -.034 | .212 | .817 | .083 |
|  | p | .231 | .471 | .000 | .146 | .000 | .828 | .167 | .000 | .599 |
| hsCRP | r | -.023 | -.056 | -.119 | -.104 | -.034 | 1.000 | .010 | .098 | .819 |
|  | p | .881 | .717 | .444 | .502 | .828 | .000 | .947 | .528 | .000 |
| lnTG | r | .902 | .145 | -.183 | .217 | .212 | .010 | 1.000 | .580 | .140 |
|  | p | .000 | .347 | .234 | .157 | .167 | .947 | .000 | .000 | .370 |
| lnTG/HDL | r | .487 | .080 | -.847 | .216 | .817 | .098 | .580 | 1.000 | .237 |
|  | p | .001 | .606 | .000 | .159 | .000 | .528 | .000 | .000 | .126 |
| lnCRP | r | .126 | -.093 | -.159 | -.226 | .083 | .819 | .140 | .237 | 1.000 |
|  | p | .422 | .554 | .310 | .146 | .599 | .000 | .370 | .126 | .000 |
| GCK-MODY | | | | | | | | | | |
|  |  | TG | TC | HDL | LDL | TG/HDL | hsCRP | lnTG | lnTG/HDL | lnCRP |
| TG | r | 1.000 | .258 | -.357 | .291 | .957 | .165 | .952 | .897 | .275 |
|  | p | .000 | .091 | .017 | .055 | .000 | .284 | .000 | .000 | .071 |
| TC | r | .258 | 1.000 | .263 | .908 | .147 | .294 | .268 | .125 | .169 |
|  | p | .091 | .000 | .084 | .000 | .340 | .053 | .079 | .418 | .271 |
| HDL | r | -.357 | .263 | 1.000 | -.076 | -.587 | .119 | -.382 | -.661 | -.001 |
|  | p | .017 | .084 | .000 | .623 | .000 | .443 | .011 | .000 | .995 |
| LDL | r | .291 | .908 | -.076 | 1.000 | .263 | .235 | .338 | .300 | .181 |
|  | p | .055 | .000 | .623 | .000 | .084 | .125 | .025 | .048 | .239 |
| TG/HDL | r | .957 | .147 | -.587 | .263 | 1.000 | .091 | .904 | .942 | .197 |
|  | p | .000 | .340 | .000 | .084 | .000 | .556 | .000 | .000 | .200 |
| hsCRP | r | .165 | .294 | .119 | .235 | .091 | 1.000 | .173 | .099 | .836 |
|  | p | .284 | .053 | .443 | .125 | .556 | .000 | .262 | .521 | .000 |
| lnTG | r | .952 | .268 | -.382 | .338 | .904 | .173 | 1.000 | .945 | .303 |
|  | p | .000 | .079 | .011 | .025 | .000 | .262 | .000 | .000 | .045 |
| lnTG/HDL | r | .897 | .125 | -.661 | .300 | .942 | .099 | .945 | 1.000 | .247 |
|  | p | .000 | .418 | .000 | .048 | .000 | .521 | .000 | .000 | .106 |
| lnCRP | r | .275 | .169 | -.001 | .181 | .197 | .836 | .303 | .247 | 1.000 |
|  | p | .071 | .271 | .995 | .239 | .200 | .000 | .045 | .106 | .000 |
| HNF1A-MODY | | | | | | | | | | |
|  |  | TG | TC | HDL | LDL | TG/HDL | hsCRP | lnTG | lnTG/HDL | lnCRP |
| TG | r | 1.000 | .372 | -.368 | .254 | .982 | -.003 | .963 | .951 | -.004 |
|  | p | . | .172 | .196 | .361 | .000 | .991 | .000 | .000 | .989 |
| TC | r | .372 | 1.000 | .232 | .950 | .267 | -.078 | .312 | .230 | -.249 |
|  | p | .172 | . | .426 | .000 | .357 | .782 | .257 | .429 | .372 |
| HDL | r | -.368 | .232 | 1.000 | .053 | -.501 | -.274 | -.361 | -.545 | -.354 |
|  | p | .196 | .426 | .000 | .857 | .068 | .343 | .205 | .044 | .214 |
| LDL | r | .254 | .950 | .053 | 1.000 | .174 | -.012 | .210 | .178 | -.160 |
|  | p | .361 | .000 | .857 | .000 | .553 | .967 | .453 | .543 | .569 |
| TG/HDL | r | .982 | .267 | -.501 | .174 | 1.000 | .086 | .916 | .919 | .084 |
|  | p | .000 | .357 | .068 | .553 | .000 | .771 | .000 | .000 | .776 |
| hsCRP | r | -.003 | -.078 | -.274 | -.012 | .086 | 1.000 | -.035 | .039 | .810 |
|  | p | .991 | .782 | .343 | .967 | .771 | .000 | .903 | .893 | .000 |
| lnTG | r | .963 | .312 | -.361 | .210 | .916 | -.035 | 1.000 | .991 | -.048 |
|  | p | .000 | .257 | .205 | .453 | .000 | .903 | .000 | .000 | .865 |
| lnTG/HDL | r | .951 | .230 | -.545 | .178 | .919 | .039 | .991 | 1.000 | -.044 |
|  | p | .000 | .429 | .044 | .543 | .000 | .893 | .000 | .000 | .881 |
| lnCRP | r | -.004 | -.249 | -.354 | -.160 | .084 | .810 | -.048 | -.044 | 1.000 |
|  | p | .989 | .372 | .214 | .569 | .776 | .000 | .865 | .881 | .000 |
| T2DM | | | | | | | | | | |
|  |  | TG | TC | HDL | LDL | TG/HDL | hsCRP | lnTG | lnTG/HDL | lnCRP |
| TG | r | 1.000 | -.040 | .323 | -.015 | .921 | .030 | .867 | .732 | .096 |
|  | p | .000 | .796 | .030 | .921 | .000 | .845 | .000 | .000 | .529 |
| TC | r | -.040 | 1.000 | .157 | -.278 | -.057 | -.120 | -.167 | -.201 | -.105 |
|  | p | .796 | .000 | .303 | .065 | .709 | .431 | .273 | .186 | .493 |
| HDL | r | .323 | .157 | 1.000 | .127 | .006 | -.185 | -.030 | -.316 | -.109 |
|  | p | .030 | .303 | .000 | .405 | .969 | .225 | .846 | .034 | .478 |
| LDL | r | -.015 | -.278 | .127 | 1.000 | -.039 | -.068 | .143 | .084 | -.005 |
|  | p | .921 | .065 | .405 | .000 | .799 | .657 | .350 | .582 | .973 |
| TG/HDL | r | .921 | -.057 | .006 | -.039 | 1.000 | .041 | .911 | .869 | .090 |
|  | p | .000 | .709 | .969 | .799 | .000 | .791 | .000 | .000 | .558 |
| hsCRP | r | .030 | -.120 | -.185 | -.068 | .041 | 1.000 | .084 | .131 | .848 |
|  | p | .845 | .431 | .225 | .657 | .791 | .000 | .582 | .393 | .000 |
| lnTG | r | .867 | -.167 | -.030 | .143 | .911 | .084 | 1.000 | .957 | .115 |
|  | p | .000 | .273 | .846 | .350 | .000 | .582 | .000 | .000 | .451 |
| lnTG/HDL | r | .732 | -.201 | -.316 | .084 | .869 | .131 | .957 | 1.000 | .139 |
|  | p | .000 | .186 | .034 | .582 | .000 | .393 | .000 | . | .362 |
| lnCRP | r | .096 | -.105 | -.109 | -.005 | .090 | .848 | .115 | .139 | 1.000 |
|  | p | .529 | .493 | .478 | .973 | .558 | .000 | .451 | .362 | . |
